# Structural color due to guided-mode resonance in silicon-on-insulator irradiated by nanosecond laser pulses

**DOI:** 10.1080/14686996.2026.2641872

**Published:** 2026-03-20

**Authors:** Vygantas Mizeikis, Cristhian Cobas Montero, Anzelms Zukuls, Kaspars Ozols, Patrik Ščajev, Yoshishige Tsuchiya, Darius Gailevičius, Daniel Moraru, Pavels Onufrijevs

**Affiliations:** a Research Institute of Electronics, Shizuoka University, Hamamatsu, Japan; b Institute of Physics and Materials Science, Faculty of Natural Sciences and Technology, Riga Technical University, Riga, Latvia; c Institute of Photonics and Nanotechnology, Vilnius University, Vilnius, Lithuania; d School of Electronics and Computer Sciences, University of Southampton, Southampton, UK; e Laser Research Center, Vilnius University, Vilnius, Lithuania

**Keywords:** structural color, guided-mode resonance, silicon-on-insulator, laser-induced periodic surface structures, laser processing, color printing

## Abstract

We demonstrate structural color generation in silicon-on-insulator wafers using nanosecond laser irradiation. Laser-induced periodic surface structures on the thin Si film act as grating couplers, enabling optical resonances that produce bright, spectrally selective structural colors at visible wavelengths. The mechanism combines grating-mediated waveguide coupling with Fabry-Perot spectral filtering, yielding optical characteristics resembling guided-mode resonance. The central wavelength is tunable across the visible spectrum by varying Si film thickness (50–70 nm range), with measured samples exhibiting green coloration at 55 nm and red at 70 nm thickness. Numerical simulations qualitatively reproduce the observed optical properties. This non-chemical, non-fading coloration offers potential applications in secure marking and process control for semiconductor manufacturing.

## IMPACT STATEMENT

Nanosecond laser-induced LIPSS enable guided-mode resonance structural color on SOI wafers by combining grating coupling and Fabry-Perot filtering for angularly stable non-chemical, maskless coloration in semiconductor security and process control

## GRAPHICAL ABSTRACT

**Figure:**
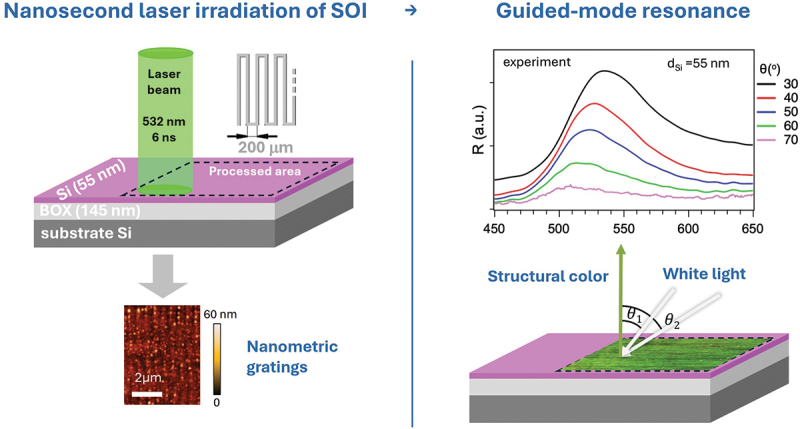

## 1. Introduction

Structural color [1] arises from light interaction with micro- and nanoscale structures through interference, diffraction, or resonance effects [2], rather than absorption or emission. Unlike conventional pigments and dyes that fade under environmental exposure, structural coloration is non-chemical and persists as long as the underlying structure remains intact. The color can be tuned across a broad spectral range by adjusting the geometry of the structure, yielding vivid angle- and polarization-dependent colors [3,4]. These unique properties make structural color materials attractive for imaging, sensing, security features, and bio-inspired coatings [5,6]. Various structures produce structural color: plasmonic nanostructures exploit localized surface plasmon resonances [7]; photonic crystals use bandgap effects and Bragg reflection [8–10]; and metasurfaces enable precise control over light amplitude, phase, and spectrum [2,11].

Many of these materials require lengthy and costly fabrication. Direct Laser Writing (DLW) technique [12,13] offers a possibility to overcome the fabrication challenge via rapid, maskless patterning of materials using the spatial translation of a laser beam. A particularly scalable approach within this domain is the generation of laser-induced periodic surface structures (LIPSS) [14,15]. Formed via self-organization mechanisms under nanosecond to femtosecond pulsed illumination on metals, semiconductors, and dielectrics [16], LIPSS generate vivid structural coloration through diffraction and interference. This versatility has enabled structural color printing [17] on Silicon-on-Insulator (SOI) substrates for semiconductor process control, anti-counterfeiting [18,19], and optical encoding [20,21]. However, the utility of standard LIPSS for secure laser marking remains limited by strong iridescence, which may limit its visual uniqueness.

Here, we demonstrate LIPSS-based laser printing on SOI wafers via a different mechanism producing vivid and comparatively less iridescent structural color. Laser-induced periodic gratings on the thin Si planar waveguide enable efficient optical coupling between free space and waveguide modes, resembling the Guided-Mode Resonance (GMR) [22,23] phenomenon observed in periodically corrugated planar waveguides and widely exploited in photonics [24]. The leaky waveguide modes responsible for optical coupling become spectrally filtered by the Fabry-Perot (FP) resonator formed by the Si planar waveguide interfaces, producing a relatively narrow optical reflectivity band at visible wavelengths and predominantly non-iridescent structural color observable at certain illumination and observation angles. The central wavelength and angular properties depend on the planar waveguide thickness and grating period. Since SOI is the staple material of the microelectronics industry and is widely used in advanced silicon photonics [25–27], the physical mechanisms outlined in this report may enable more secure color laser marking of SOI and other thin planar waveguide-based devices for process control and anti-counterfeiting.

## 2. Experimental details

**Figure 1.**
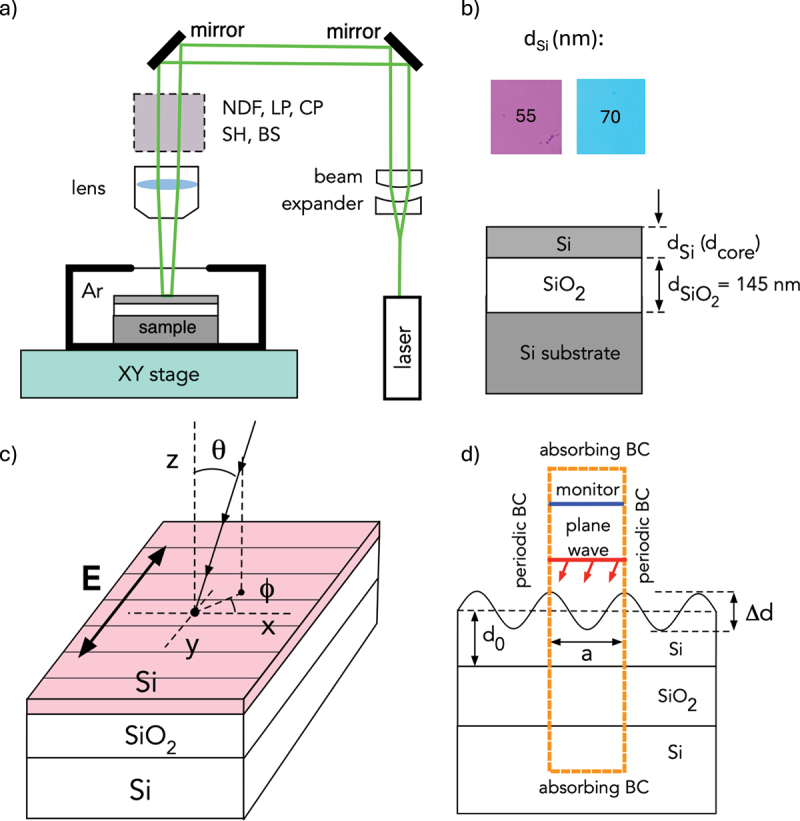
(a) Opto-Mechanical setup for laser processing. Gray-shaded box represents optical components employed to control laser irradiation conditions: NDF – neutral density filter, LP, CP – linear and circular polarizers, respectively, SH – shutter; (b) geometry and parameters of SOI substrates and their colors; (c) geometry of sample reflectance measurements, with orientation of laser-induced gratings on the top Si surface shown by thin solid lines; (d) model structure and geometry for FDTD simulations.

The opto-mechanical setup for laser processing is shown in Figure 1(a). We employed the second-harmonic output of a Nd:YAG laser (NL301G, Ekspla, Lithuania) operating at $\lambda_{L}=532$ nm with 4 ns pulse duration and 10 Hz repetition rate. At the sample plane, the laser beam had approximately 1 mm diameter with a top-hat spatial intensity profile. Samples were translated at a constant velocity of 0.5 mm/s and scanned along parallel lines separated by 0.2 mm to achieve uniform patterning. The sample was mounted in a hermetic cell filled with argon gas at 1.1 atm to minimize oxidation. The laser power density was adjusted to approximately 43.3 MW/cm${\;}^{2}$ to maximize the observable structural color.

The schematic geometry, structural parameters, and color of pristine SOI wafers are shown in Figure 1(b). The wafers were commercially obtained from Soitec and had two different thickness of the top Si layer, $d_{\mathrm{Si}}=55\;$ nm and 70 nm, and the same thickness of the buried oxide (BOX) layer $d_{SiO_{2}}=145\;$ nm. The first group of wafers appeared pink under white-light illumination, while the second group exhibited a blue-green appearance. Top Si layer was weakly doped by boron and had the specific resistance of (8.5∼11.5)Ω⋅ cm. Prior to laser processing, the samples were diced into chips having the size of about (2×4) cm${\;}^{2}$. An initial cleaning process typically consists of ultrasonication in acetone and ethanol, followed by three sequences of cleaning in a mixture of sulfuric acid and hydrogen peroxide (H${\;}_{2}$SO${\;}_{4}$:H${\;}_{2}$O${\;}_{2}$ = 4:1) and etching of the top oxide layer in a diluted hydrofluoric acid (HF:H${\;}_{2}$O = 1:20). According to ellipsometry and step-profiler measurements performed on wafers prepared for the fabrication of electronic nanodevices, surface roughness of pristine wafers was within approximately 0.2 nm. The samples were observed using an optical microscope (VK-X200, Keyence), a scanning electron microscope (SEM, JSM-7600F, JEOL). Their surface morphology was analyzed by atomic force microscope (AFM, Smena, NT-MDT) operated in semi-contact mode with a gold-coated silicon NSG03 probe (NT-MDT). Surface was characterized by scanning areas of (10×10)μm${\;}^{2}$ at a velocity of 20.2μm/s.

The optical setup geometry for reflectivity measurements is illustrated in Figure 1(c). Samples were imaged using an optical microscope with an objective lens of numerical aperture NA =0.3. A halogen lamp equipped with a fiber bundle and collimating lens produced spectrally broadband incident light with a divergence angle of approximately ±25deg. The average incidence angle θ (Figure 1(c)) was adjustable within θ=30–75deg. Azimuthal sample orientation angle ϕ, defined with respect to the average orientation of LIPSS grating ridges could be varied continuously in the range ϕ=0–360deg. The reflected light spectrum was analyzed using a fiber-coupled compact spectrometer.

**Figure 2.**
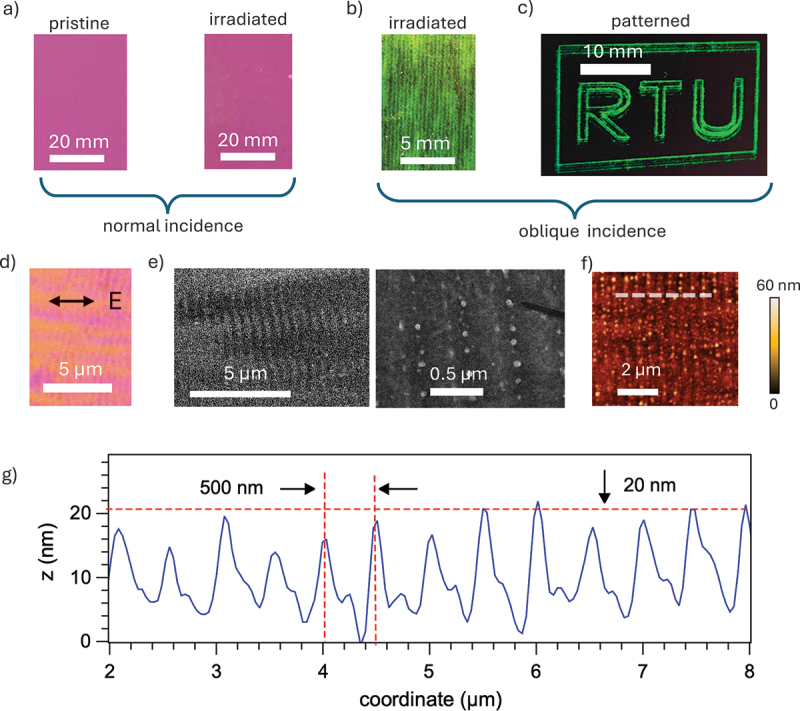
(a) Color of pristine and laser-irradiated SOI areas at normal incidence; (b) green structural color of laser-irradiated SOI area seen at illumination and observation angles of about 60°; (c) demonstration of color laser marking on Si surface using DLW; (d–f) laser-processed SOI surface images taken by (d) optical microscopy (orientation of the linear polarization of laser pulses is indicated by the arrow), (e) SEM, and (f) AFM; (g) detailed AFM profile measured along the gray dashed line in (f) with estimates of the grating period and maximum height modulation. All data are for samples with $d_{\mathrm{Si}}=55$ nm.

Theoretical simulations of reflectivity spectra were performed using the finite-difference time-domain (FDTD) technique (Ansys Lumerical FDTD). The geometry is shown in Figure 1(d). A broadband linearly polarized plane wave was launched toward the sample surface at incidence angles defined by θ and ϕ, corresponding to experimental measurements. The LIPSS gratings on the top Si layer were represented as ideal sinusoidal surface relief gratings with amplitude and period close to experimental values. Finer surface features (e.g. spherical nanoparticles and surface roughness, see Figure 2(e,f)) were ignored. The reflected field was recorded by a virtual monitor and transformed to the far-field to reveal diffracted orders, enabling evaluation of polarization-averaged spectra. Periodic boundary conditions were applied in the xy-plane, while perfectly matched layer (PML) boundaries were used along the z-axis to suppress artificial reflections. Dispersive optical properties of the constituent materials were included using the built-in material database.

## 3. Results and discussion

### 3.1. Observable sample color and surface morphology

Visual inspection provides the first evidence of the distinct structural color mechanism. Figure 2(a–c) summarizes colors of pristine and laser-processed samples, while Figure 2(d–f) shows detailed surface structure. At normal incidence, samples with $d_{\mathrm{Si}}=55$ nm exhibit nearly identical pink color, regardless of laser irradiation (Figure 2(a)).

At oblique angles θ=30–70deg, laser-processed samples with $d_{\mathrm{Si}}=55$ nm exhibit bright green and green-blue coloration (Figure 2(b)), whereas pristine regions remain dark. The color is relatively angle-independent, but its brightness varies with illumination and observation angles. Under normally-incident white light, a vivid structural color can be observed along two symmetric directions at θ=±60deg. Under oblique illumination at θ=60 and ϕ=±90deg, a single colorful beam counter-propagating toward the source is observed. These angular dependencies suggest diffraction by laser-generated surface gratings plays a key role. Samples with $d_{\mathrm{Si}}=70$ nm exhibited red structural color, suggesting the role of Fabry-Perot modes. The practical utility of this stable coloration is illustrated in Figure 2(c), which demonstrates color laser patterning using DLW.

Surface morphology of laser-irradiated SOI substrates is presented in Figure 2(d–f). Periodic patterns revealed by optical microscopy (Figure 2(d)) indicate surface gratings with average orientation perpendicular to the laser polarization – a characteristic feature of LIPSS [16]. SEM images (Figure 2(e)) reveal grating periods of a=500–600 nm and surface grooves and ridges decorated with quasi-periodic linear arrays of nanoparticles with diameters ≤50 nm.

AFM imaging (Figure 2(f)) shows quasi-harmonic surface profiles with maximum height modulation of ≈20 nm. Nanoparticles decorating the gratings extend to a maximum height of about 60 nm. Wide-area AFM images indicate spatial variation of the grating period within a=500–650 nm. These features suggest LIPSS formation via laser-induced photothermal effects, such as Si melting, evaporation, and recrystallization. Since these and other possible modification mechanisms may also lead to chemical modifications of the surface, resulting in hydrocarbon contamination and generation of defects that may generate the conventional non-structural coloration via light absorption and emission. This possibility was examined using contact angle measurements in water and energy-dispersive X-ray spectroscopy (EDS). No major modification was found. The results indicate the absence of non-structural coloration in our samples, and are presented in the Supplementary Information (SI).

To contextualize these morphological features, it is instructive to compare them with prior studies on similar material systems. Recent work on LIPSS in thin amorphous Si films using UV nanosecond laser irradiation [28] reported similar features: LIPSS period close to the wavelength, grating orientation orthogonal to laser polarization, and linear chains of nanoparticles decorating the gratings. Although structural color was not their focus, weak spectral signatures of GMR in transmission at normal incidence were observed. Generation of deep and highly regular periodic LIPSS gratings having a harmonic surface profile was reported recently [29] in SOI structures having somewhat thicker top Si $d_{\mathrm{Si}}=200\;$ nm irradiated by a femtosecond laser pulses. These structures were found to exhibit the conventional iridescent coloration due to the grating dispersion and were intended to be applied as grating couplers, since signatures of guided modes at near-infrared wavelengths were found using numerical simulations. This result implies the presence of GMR, but its possible effect on the structural color could not be observed outside the visible spectral range. In contrast, our SOI samples with thinner waveguide highlight the unique possibility to enhance the reflectivity at visible wavelengths and realize the structural color via GMR mechanism.

GMR occurs in periodically corrugated planar waveguides when externally incident waves are diffracted and phase-matched with waveguide modes, enabling coupling between free space and the waveguide. GMR is widely exploited in photonic devices [30,31], enabling extremely narrow-band spectral filters [32], high-quality resonators [33], wide-band reflectors [34], and optical bound states in the continuum [35]. Since the top Si film in our samples is a periodically corrugated planar waveguide, the observed spectral and angular features tentatively relate to GMR.

### 3.2. Angle-dependent reflectivity spectra

Grating-mediated optical coupling depends critically on wave-vector conservation. We performed angle-dependent optical reflectivity measurements using broadband, unpolarized incident light beams – conditions representing typical structural color observation by the naked eye.

In our experimental geometry (Figure 1(c)), wave-vector components of incident light are:

(1) $$k_{x}=k_{0}\sin\theta \cos\phi ;\;k_{y}=k_{0}\sin\theta \sin\phi ;\;k_{z}=k_{0}\cos\theta ,$$

where $k_{0}=2\pi /\lambda_{0}$ is the wave-vector magnitude in air. Since the grating vector is oriented along the y-axis, wave vector component $k_{y}$ is modified by the grating. In-plane phase-matching conditions between the incident wave and diffracted orders are:

(2) $$k_{x}^{out}=k_{x}^{in}=k_{0}\sin\theta \cos\phi$$

$$k_{y}^{out}=k_{y}^{in}+m(2\pi /a)=k_{0}\sin\theta \sin\phi +m(2\pi /a),$$

where m=0,±1,±2,… is the diffraction order. For a grating period a=500–650 nm, wavelength 500–540 nm (where structural color is observed), illumination at θ≈60deg and ϕ=±90deg (optimum for strongest coupling), m=0 specular reflection and m=−1 diffracted order are confirmed. The diffracted order propagation angle, θ=0–14deg, falls within the acceptance angle of the microscope lens.

Excitation of guided planar waveguide modes via diffraction must satisfy phase matching:

(3) $$x-\mathrm{axis}:\;k_{0}\sin\theta \cos\phi =0,$$

$$y-\mathrm{axis}:\;k_{0}\sin\theta \sin\phi +m(2\pi /a)=k_{0}n_{\mathrm{eff}},$$

where $n_{\mathrm{eff}}$ is the effective refractive index of the mode. Modes of thin waveguides with d≪λ are weakly confined, with fields predominantly concentrated outside the high-index waveguide; therefore $n_{\mathrm{eff}}\to n_{\mathrm{air}}=1$ or $n_{\mathrm{Si}O_{2}}\approx 1.5$. According to Equations (2) and (3), deviation from the optimum azimuthal angle ϕ=±90deg increases the component $k_{x}$ parallel to the grating lines, violating phase-matching and suppressing reflectivity. The incidence angle θ controls $k_{y}$, which should lead to wavelength dependence of diffraction angle producing iridescent structural color – however, this conventional iridescence was not observed in practice.

**Figure 3.**
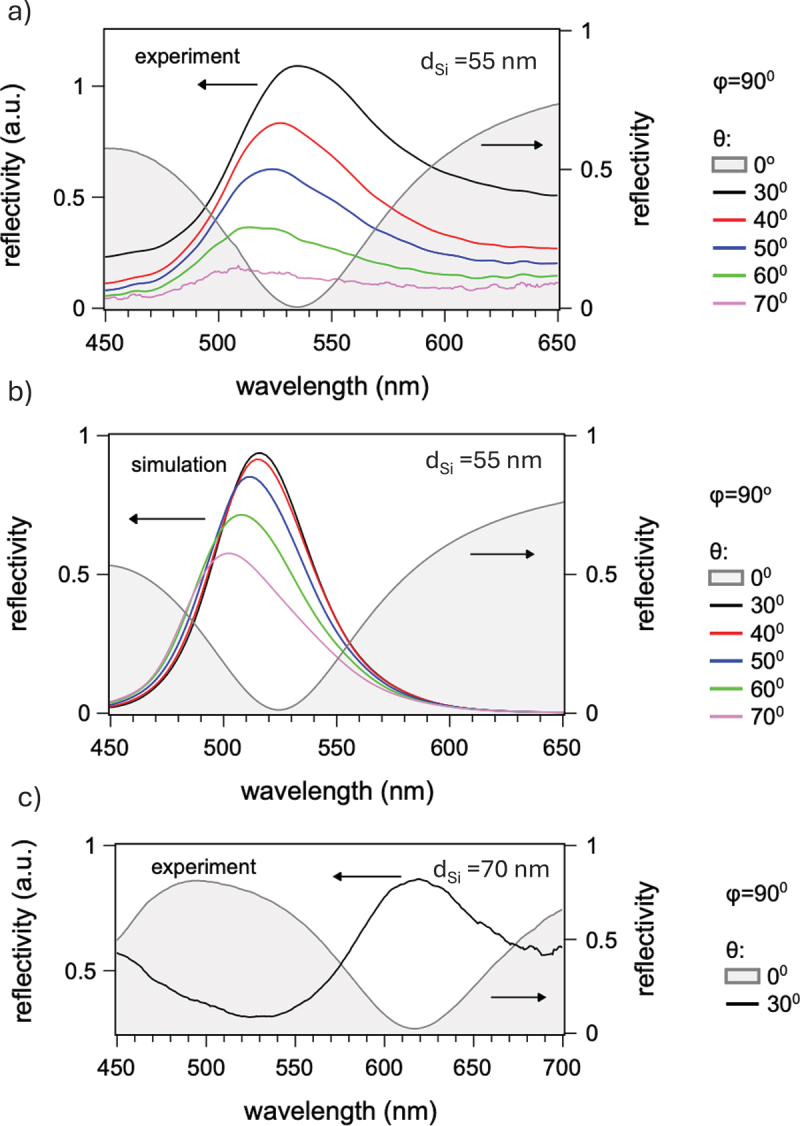
(a) Measured reflectivity spectra of SOI sample with $d_{\mathrm{Si}}=55$ nm; (b) corresponding reflectivity spectra simulated by FDTD; (c) measured reflectivity spectra of SOI sample with $d_{\mathrm{Si}}=70$ nm.

To quantify these visual observations, we performed angle-dependent spectral analysis. Figure 3(a) shows measured reflectivity spectra of the sample with $d_{\mathrm{Si}}=55$ nm at different incidence angles θ and fixed azimuthal angle ϕ=90deg.

As a reference, the spectrum at normal incidence exhibits a strong reflectivity dip centered at λ=535nm, originating from destructive interference between waves reflected at air/Si and Si/SiO${\;}_{2}$ interfaces. Its spectral position is determined by the Si film thickness $d_{\mathrm{Si}}$. Pink color at normal incidence results from spectral convolution between broadband incident light and two high-reflectivity regions on both sides of the dip.

Pristine and laser-processed areas show nearly identical reflectivities at normal incidence. However, at oblique illumination, laser-processed samples exhibit a pronounced reflectivity peak beginning at $\theta_{\min}=30\deg$. Its intensity gradually decreases with angle, while its spectral peak, which initially coincides with the dip at normal incidence, exhibits a weak blue shift with increasing θ. In this and subsequent figures, reflectivity spectra measured at oblique incidence were obtained by normalizing raw spectrum of the reflected wave to that of the incident wave measured at normal incidence. This approach produces correct spectral shape of the reflectivity, but neglects its absolute amplitude. Hence, arbitrary units are used for vertical axis in the relevant plots. Absolute reflectivity is estimated at ≈0.1 for θ=60deg.

The blue shift of the reflectivity peak with increasing θ is indicative of momentum conservation in the periodic structure, requiring a shorter incident wavelength (larger momentum). While qualitatively consistent with Equations (1–3), the measured blue shift of about 30 nm is surprisingly small compared to a strong dispersive color change expected from Equation (2) and seen experimentally in various gratings, including highly regular LIPSS patterns induced by femtosecond laser irradiation of SOI substrates [29]. This limited dispersive shift is responsible for the relative purity and angular stability of the structural color described in Section 3.1.

Figure 3(b) shows FDTD-simulated spectra using sample parameters and measurement angles matching experiments. The LIPSS structure was approximated by a sinusoidal surface relief grating with period a=500 nm and total modulation depth of 10 nm. Reflectivity spectra of the m=−1 diffracted order were evaluated using polarization averaging.

The simulated spectrum at normal incidence θ=0deg closely resembles the experimental spectrum. Spectra for oblique incidence qualitatively reproduce essential features (central wavelength, spectral bandwidth, blue shift with θ) of the experimental spectra. Figure 3(c) shows reflectivity spectra for the SOI structure with a thicker top Si layer, $d_{\mathrm{Si}}=70$ nm. At normal incidence, this structure exhibits a reflectivity dip centered at 615 nm. Its blue-green appearance relates to the high-reflectance band centered at 500 nm. Under oblique illumination, the laser-irradiated sample exhibits a reflection band centered at 615 nm.

The data illustrate two general trends governing the color generation. First, the reflectivity peak at oblique incidence emerges at the same wavelength as the reflectivity dip at normal incidence. Second, its central wavelength is approximately proportional to the top Si waveguide thickness $d_{\mathrm{Si}}$. It can be extrapolated that the full color range can be realized for $d_{\mathrm{Si}}\approx 50$–80 nm in different SOI wafers, or in the same SOI wafer by locally thinning its top Si film, for instance, by laser polishing [36].

**Figure 4.**
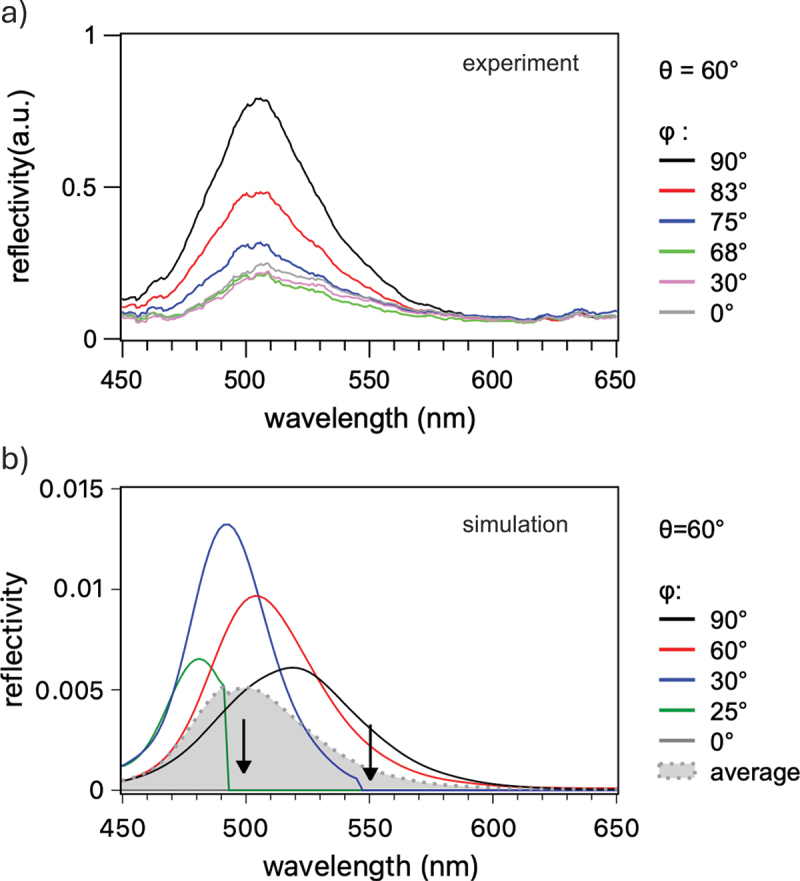
(a) Measured reflectivity spectra of SOI sample with $d_{\mathrm{Si}}=55$ nm for different azimuthal illumination angles; (b) corresponding reflectivity spectra simulated by FDTD (different azimuthal angles were used compared to experiments illustrate the qualitative behavior).

Azimuthal dependencies of measured and simulated reflectivity spectra are shown in Figure 4. In experimental spectra (Figure 4(a)), the reflectivity band becomes progressively suppressed as angle ϕ deviates from the optimum value, while its center wavelength remains fixed at approximately 510 nm. Simulated spectra (Figure 4(b)) exhibit different behavior. As ϕ decreases from 90deg to 0deg, variation of both magnitude and spectral position is observed, with a relatively small blue shift of about 40 nm within the full azimuthal angular range. At angles ϕ≤30deg, a pronounced cut-off marks the wavelength above which the diffracted order becomes non-propagating. Complete suppression of reflectivity is seen at ϕ=0deg.

The data in Figure 4 indicate that the theoretical model ignores some essential features of real samples. As seen in Figure 2(d,e), the LIPSS grating orientation is inhomogeneous. Consequently, reflectivity measurements integrate spectra from multiple regions with different azimuthal angles ϕ. This is supported by the experimental result showing finite reflectivity even at ϕ=0deg. As a crude approximation, non-uniformity may be represented by averaging spectra within the full angular range ϕ=0–90deg. The result is shown in Figure 4(b) by the gray-shaded area, which has a shape similar to the experimental spectra in Figure 4(a). Future improvements to FDTD simulations could include surface roughness, nanoparticle arrays, and angular divergence of illuminating light.

### 3.3. Structural color and guided-mode resonance

**Figure 5.**
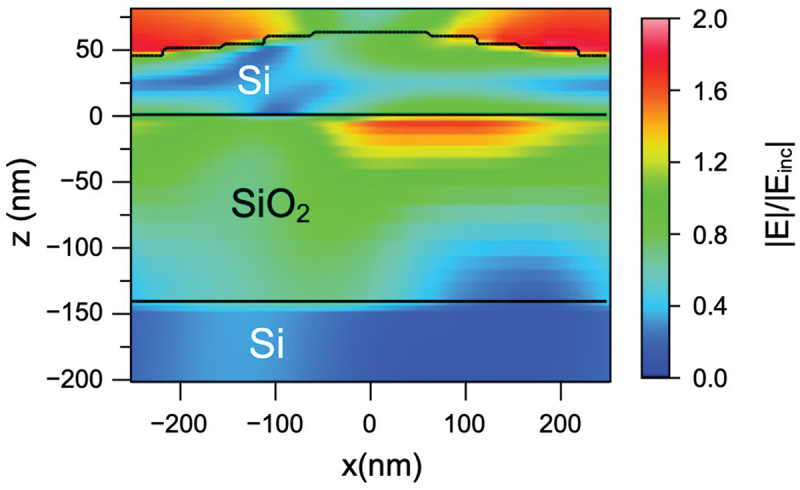
Simulated spatial distribution of the normalized electric field amplitude in the SOI structure at λ=500 nm, θ=60, ϕ=90 for an incident wave polarized along the x-axis (parallel to the grating lines). Dark lines emphasize the boundaries of the Si film. The spatial region spanning one grating period is shown.

The influence of the planar waveguide mode cannot be demonstrated experimentally, as this would require near-field mapping of the waveguide region with high spatial resolution. Hence, we use FDTD simulations for theoretical visualization. Figure 5 shows the simulated cross-sectional view of the field distribution in the y-z plane at resonance under oblique illumination (θ=60deg, ϕ=90deg). The field amplitude $\left|E\right|$ is normalized to the incident field amplitude $\left|E_{\mathrm{inc}}\right|$. Si is strongly absorbing at visible wavelengths, with an absorption coefficient α=9340 cm${\;}^{-1}$ at 550 nm [37] suggesting a short propagation length in bulk Si for visible light: $l_{\mathrm{prop}}\approx 0.5$ μm, comprising approximately one LIPSS period. In a thick waveguide modulated by a grating, strong mode confinement would result in fast extinction due to absorption and suppression of diffractive mechanisms. However, in a thin waveguide, the overlap of the guided modes with Si layer becomes dramatically reduced, leading to a reduced absorption and an increased propagation length. From the calculated transverse field distribution in Figure 5, the mode confinement factor Γ≈0.12 was estimated, meaning that only about 12% of its power is concentrated in the strongly absorbing Si layer, while the major part resides in the superficial air and underlying SiO${\;}_{2}$ regions. This leads to a reduced effective absorption coefficient αΓ and an increased light propagation length $l_{\mathrm{prop}}\approx 4.5\;$ μm. During the propagation, weakly confined waveguide modes leak to free space along the directions determined by diffraction from LIPSS gratings, thus resulting in an angular selectivity for the observation of structural color. It should be noted that this leakage is likely to increase losses and reduce the propagation length. From the spectral width of the measured reflectivity peaks Δλ≈25−50 nm, and the corresponding Q factor of the resonance Q≈15, one can deduce the propagation length of $l_{\mathrm{prop}}\approx 0.75$ μm, i.e. close to the Si bulk value. However, this loss mechanism is not detrimental but functional, representing desired out-coupling of resonant light toward the detector; the short propagation length manifests via the spectral bandwidth, but does not suppress GMR mechanism.

Spectral purity of the structural color in our SOI structures can be attributed to the low waveguide thickness. In thick waveguides with d>λ/2, the grating mainly determines the resonance condition and establishes a strong relationship between the grating period a and the wavelength λ according to Equations (1–3). This is expected from conventional GMR models [22,32,38]. In thin waveguides with d≪λ/2, the grating provides coupling to the waveguide, whereas the resonance condition is determined by the vertical FP cavity between the top and bottom interfaces of the planar waveguide. FP cavity resonance in nanostructured Si and SiO${\;}_{2}$ metasurfaces has been employed to create and enhance the structural color [20,39]. In our study, the conclusion about the crucial role of FP cavity resonance is supported by the observation of spectral matching between the reflectivity dip at normal incidence and the reflectivity peak at oblique incidence in Figure 3(a,c). In these circumstances, the roles of the grating and waveguide become somewhat decoupled, leading to deviation from the commonly accepted GMR mechanism. Nevertheless, because the waveguide mode and grating play crucial roles, it justifies the use of the term “guided-mode resonance” to describe the structural color.

Finally, it is relevant to comment briefly on the shape asymmetry of reflectance bands seen in the measured and simulated reflectivity spectra in Figures 3 and 4. Asymmetric peaks with tails extending into long wavelengths are indicative of Fano resonance caused by interference between spectrally narrow and a spectrally broad radiation sources. Tentatively, we can ascribe the former to the GMR resonance and FP cavity, and the latter to the conventional diffraction from the LIPSS grating. Detailed analysis of this effect goes beyond the scope of this study and will be addressed in future.

## 4. Conclusions

We have demonstrated structural color generation in silicon-on-insulator wafers through nanosecond laser irradiation. Laser-induced periodic surface structures on the thin Si film create grating couplers that enable efficient optical coupling between free space and waveguide modes, producing optical characteristics resembling guided-mode resonance. The resulting angle-dependent reflection exhibits bright, spectrally selective structural color tunable across the visible spectrum through Si film thickness variation (50–70 nm range). Practically, it defines the convenient SOI top film thicknesses for single-step maskless color marking.

The observed behavior differs from conventional diffraction gratings through reduced angular dispersion and enhanced spectral selectivity. This arises from the interplay between grating-mediated coupling and Fabry-Perot resonances in the Si layer, where the thin waveguide geometry causes the resonance condition to be determined primarily by the vertical cavity rather than the grating period. Despite strong absorption in Si at visible wavelengths, the predominantly evanescent character of the guided mode in the Si layer enables effective light propagation over tens of micrometers.

This non-chemical, non-fading coloration mechanism offers potential applications in secure marking and authentication of semiconductor components. Compatibility with direct laser writing enables maskless patterning for process control and anti-counterfeiting. Future work will pursue refinement of the theoretical model, including surface morphology statistics and rigorous GMR analysis, to better understand the coupling mechanisms and optimize the structural color characteristics.

## Supplementary Material

Supplemental Material
