# Supplementary material for: Structural color due to guided-mode resonance in silicon-on-insulator irradiated by nanosecond laser pulses
Source: Sci Technol Adv Mater. 2026 Mar 20;27(1):2641872. doi: 10.1080/14686996.2026.2641872 (PMC13040576; doi:10.1080/14686996.2026.2641872)
Supplement: Supplemental Material [file TSTA_A_2641872_SM8347.docx]

Supporting Information

Structural color due to guided-mode resonance in silicon-on-insulator irradiated by nanosecond laser pulses

Vygantas Mizeikis^a^, Cristhian Cobas Montero^b^, Anzelms Zukuls^b^, Kaspars Ozols^b^, Patrik Ščajev^c^, Yoshishige Tsuchiya^d^, Darius Gailevičius^e^, Daniel Moraru^a^, Pavels Onufrijevs^b^

^a^Research Institute of Electronics, Shizuoka University, Hamamatsu, Japan; ^b^Institute of Physics and Materials Science, Faculty of Natural Sciences and Technology, Riga Technical University, Riga, Latvia; ^c^Institute of Photonics and Nanotechnology, Vilnius University, Vilnius, Lithuania; ^d^Laser School of Electronics and Computer Sciences, University of Southampton, Southampton, United Kingdom; ^e^Laser Research Center, Vilnius University, Vilnius, Lithuania

*Corresponding author: Pavels Onufrijevs, Ph.D.

Institute of Physics and Materials Science, Faculty of Natural Sciences and Technology, Riga Technical University, Riga, Latvia.

E-mail: [Pavels.Onufrijevs@rtu.lv](mailto:Pavels.Onufrijevs@rtu.lv)

During the laser processing inert gas ambient atmosphere was used instead of air, in order to prevent carbon contamination from CO_2_. This may lead to the Si surface doping by carbon[1], which could be undesirable contamination in our study.

Static water contact angle measurements were used to assess possible surface-chemical changes induced by laser processing. The results are provided in Fig.S1. Bulk Si and pristine SOI samples exhibited contact angles of ~60–63°, while laser-irradiated SOI showed a moderate decrease to ~55°. This behavior indicates a slight increase in surface polarity after irradiation, likely due to slight oxidation. Alcohol cleaning resulted in only a minor additional reduction. Overall, these results suggest that surface chemistry changes are limited and play a secondary role compared to structural effects.

**Figure S1.** Static water contact angles measured on bulk Si, pristine SOI, laser-irradiated SOI, and laser-irradiated SOI after alcohol cleaning. Error bars represent standard deviation.

As a further check of surface chemical changes, Energy-Dispersive X-ray Spectroscopy measurements (EDS) were performed using JSM-7001F electron microscope operating at 10kV accelerating voltage, and equipped with Bruker XFlash Detector 5010. The results are summarized in Fig. S2. The images in the Figure were taken in laser-irradiated areas, images of pristine areas are not shown. In the images, spatial distributions of oxygen (O), carbon (C), and silicon (Si) are completely random and uncorrelated with periodic and non-periodic features seen in the secondary electron (SE) image. Importantly, there is no sign of carbon contamination on the surface. Si to O ratio in pristine and laser-irradiated areas show only minor decrease, most likely reflecting laser-induced oxidation. Hence, the most likely chemical change is the oxidation of Si, depletion of oxygen in the subsurface insulator SiO_2_ layer, or in the thin natural oxide film on the top of Si waveguide.

In conclusion, both the contact angle and EDS measurements do not indicate significant surface contamination that may result in non-structural color.


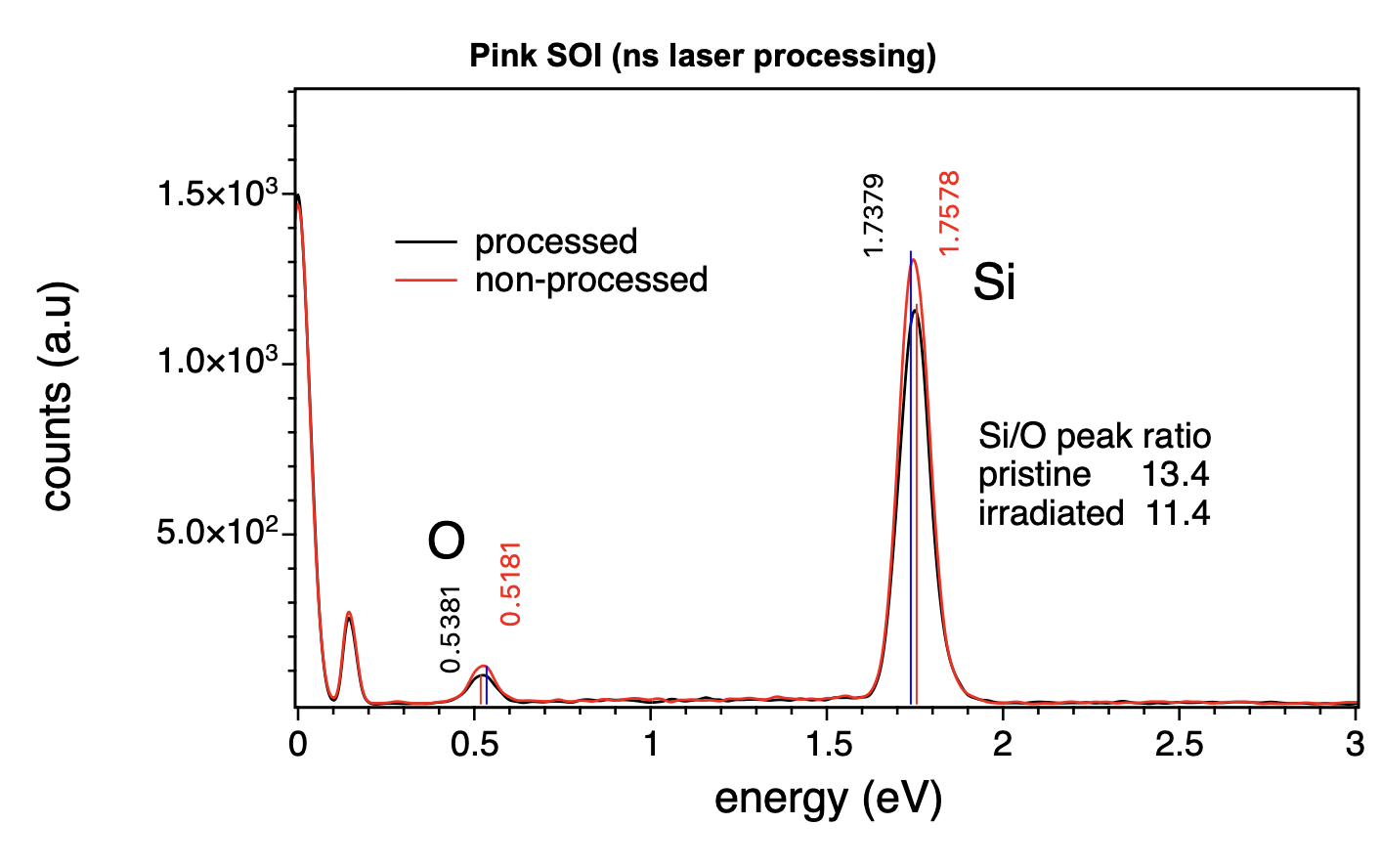

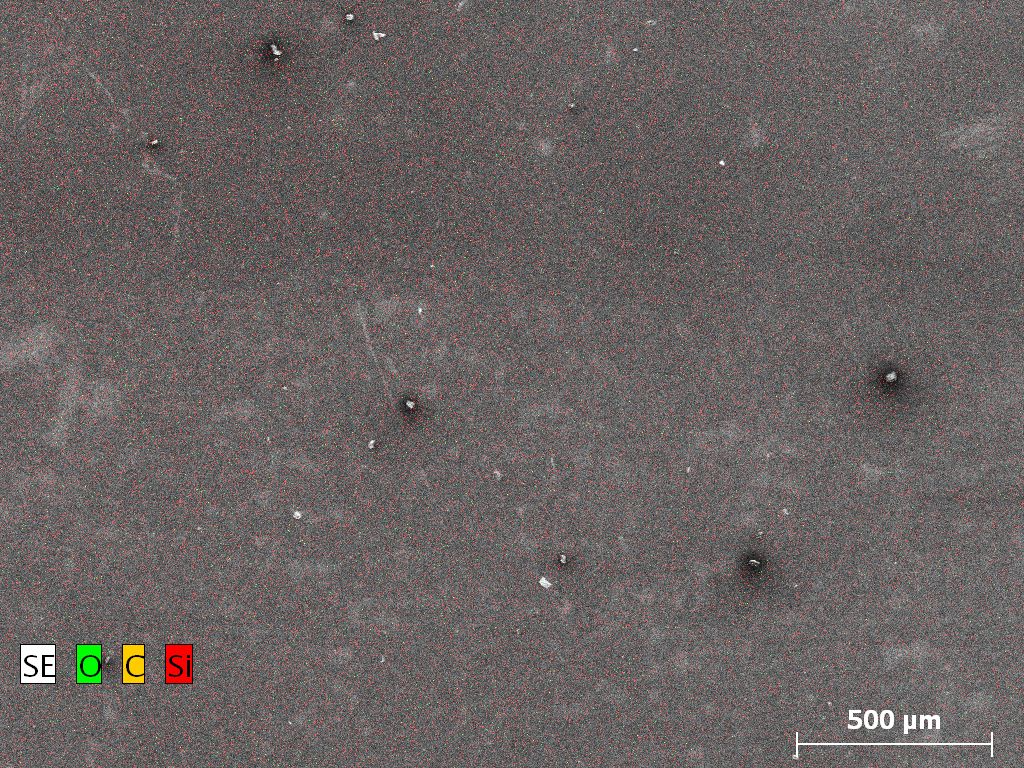

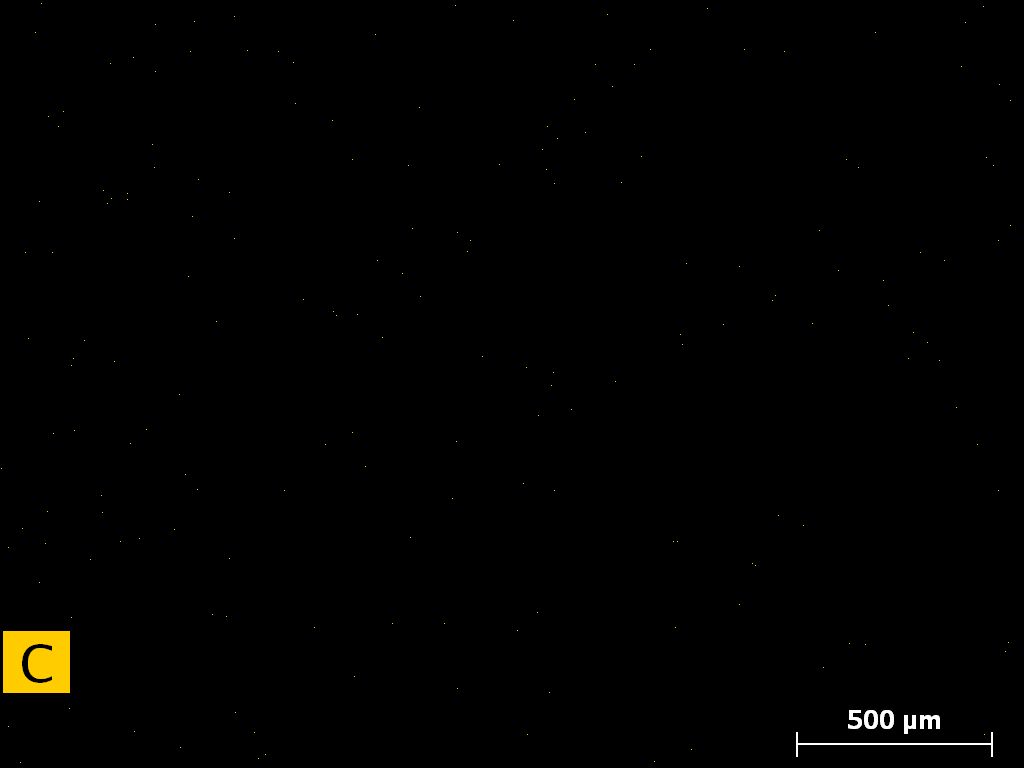

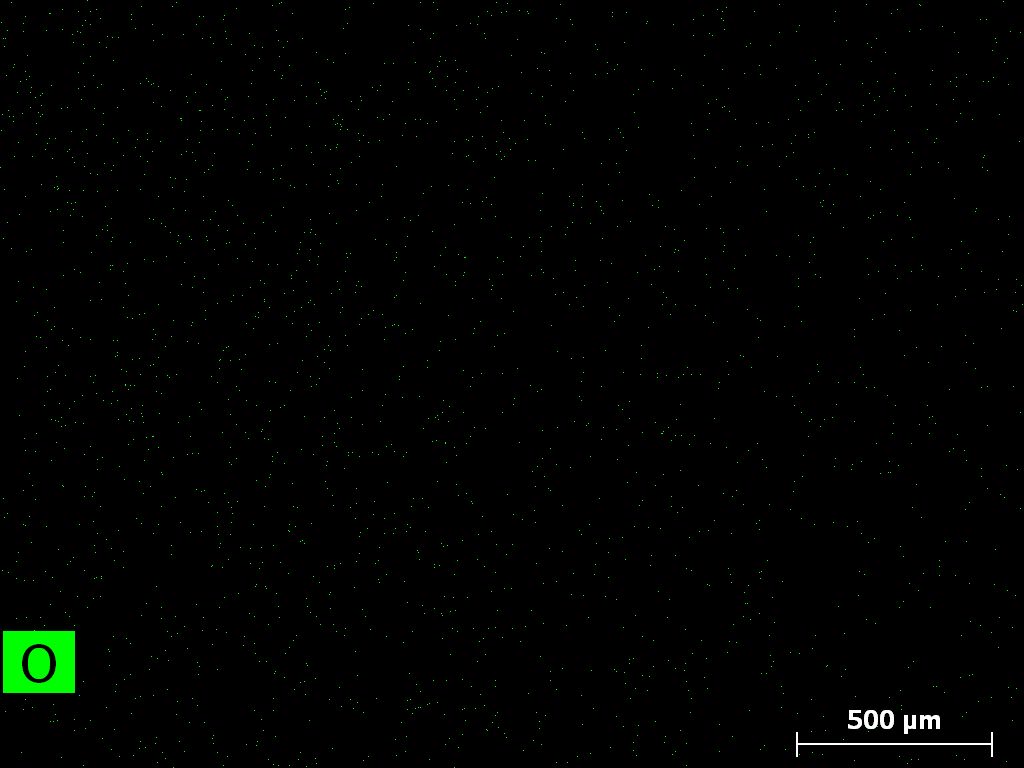

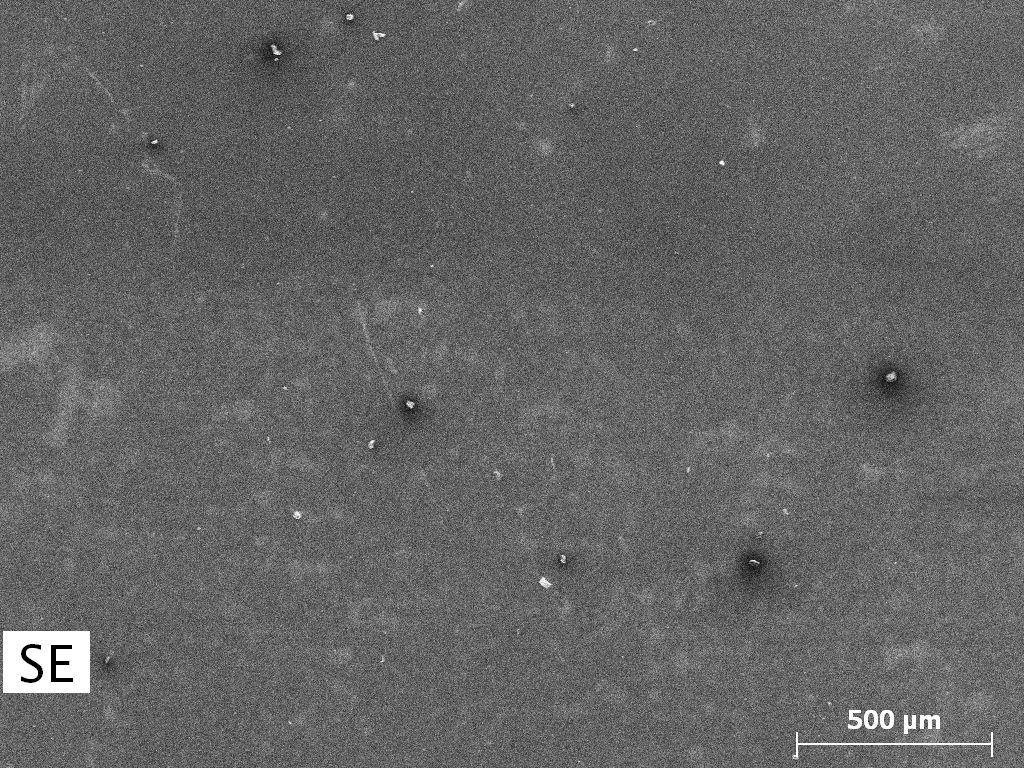

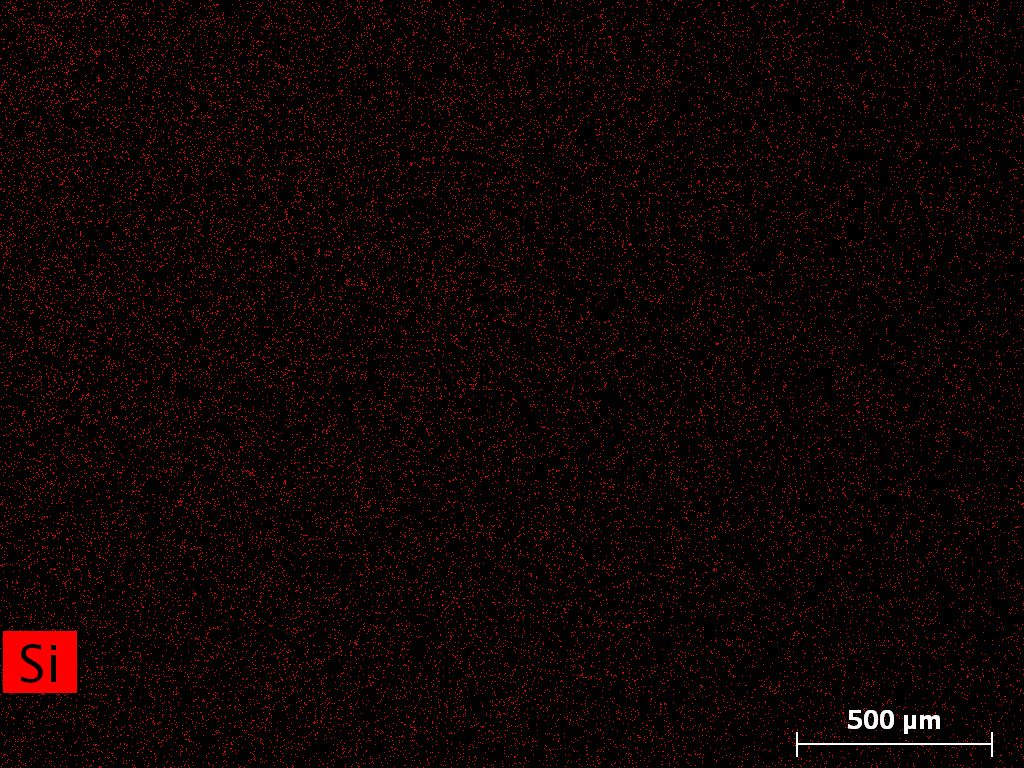

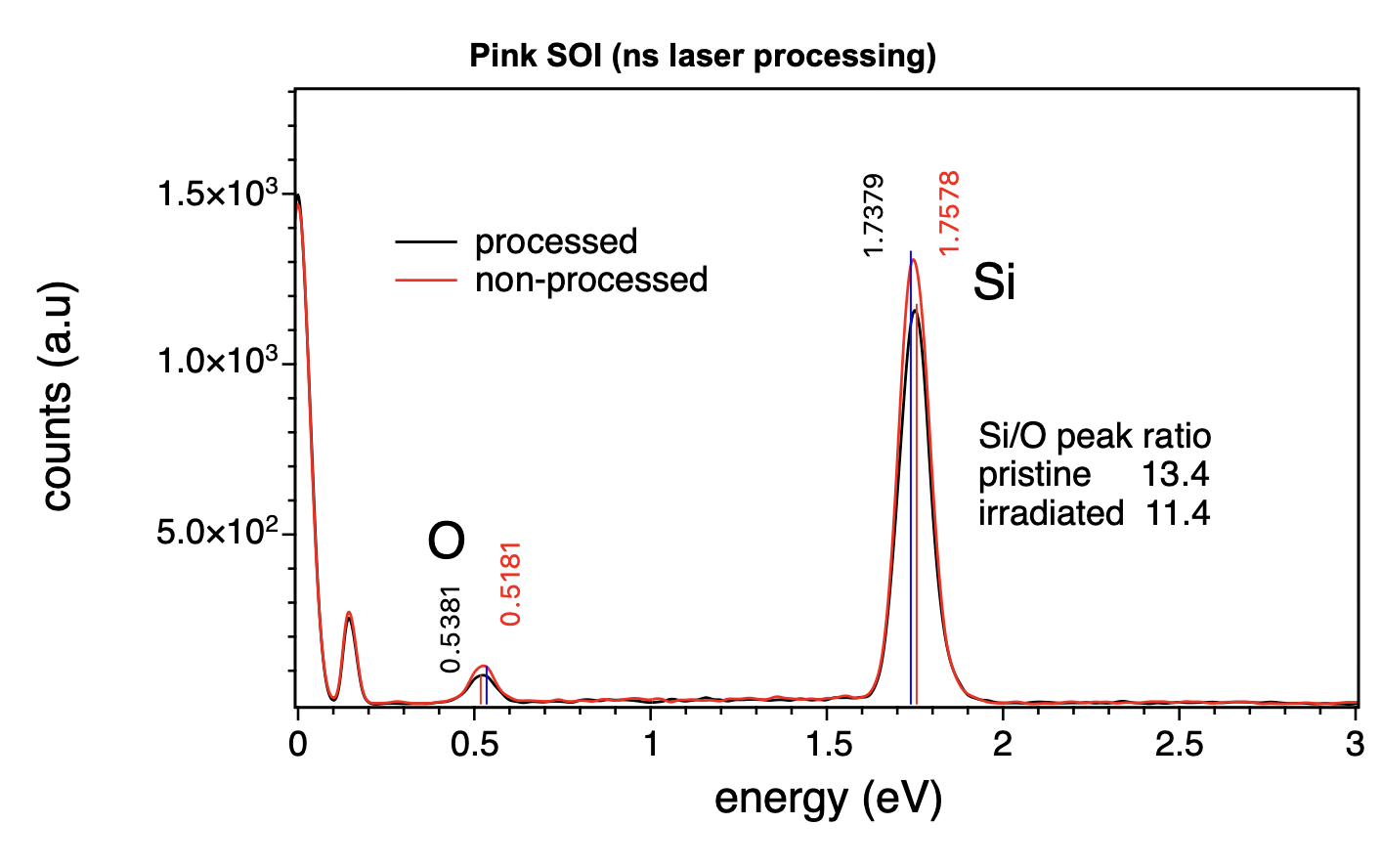


**Figure S2.** Energy-Dispersive X-ray Spectroscopy (EDS) analysis results. Images show SE, O, C, and Si intensity distributions. The graph shows EDS spectra for the pristine and irradiated areas, and an estimate of Si to O ratio.

**References**

[1] Raciukaitis G, Brikas M, Kazlauskiene V, Miskinis J. Doping of silicon by carbon during laser ablation process. *J Phys Conf Ser*. 2007;59(1):150. doi:10.1088/1742-6596/59/1/032.
